# Quantitative Trait Loci for Mercury Accumulation in Maize (*Zea mays* L.) Identified Using a RIL Population

**DOI:** 10.1371/journal.pone.0107243

**Published:** 2014-09-11

**Authors:** Zhongjun Fu, Weihua Li, Qinbin Zhang, Long Wang, Xiaoxiang Zhang, Guiliang Song, Zhiyuan Fu, Dong Ding, Zonghua Liu, Jihua Tang

**Affiliations:** 1 National Key Laboratory of Wheat and Maize Crops Science, Collaborative Innovation Center of Henan Grain Crops, College of Agronomy, Henan Agricultural University, Zhengzhou, China; 2 Maize Research Institute, Chongqing Academy of Agricultural Sciences, Chongqing, China; CRG, Spain

## Abstract

To investigate the genetic mechanism of mercury accumulation in maize (*Zea mays* L.), a population of 194 recombinant inbred lines derived from an elite hybrid Yuyu 22, was used to identify quantitative trait loci (QTLs) for mercury accumulation at two locations. The results showed that the average Hg concentration in the different tissues of maize followed the order: leaves > bracts > stems > axis > kernels. Twenty-three QTLs for mercury accumulation in five tissues were detected on chromosomes 1, 4, 7, 8, 9 and 10, which explained 6.44% to 26.60% of the phenotype variance. The QTLs included five QTLs for Hg concentration in kernels, three QTLs for Hg concentration in the axis, six QTLs for Hg concentration in stems, four QTLs for Hg concentration in bracts and five QTLs for Hg concentration in leaves. Interestingly, three QTLs, *qKHC9a*, *qKHC9b*, and *qBHC9* were in linkage with two QTLs for drought tolerance. In addition, *qLHC1* was in linkage with two QTLs for arsenic accumulation. The study demonstrated the concentration of Hg in Hg-contaminated paddy soil could be reduced, and maize production maintained simultaneously by selecting and breeding maize Hg pollution-safe cultivars (PSCs).

## Introduction

Mercury (Hg) is a non-essential element in higher plants, and is one of the most hazardous heavy metals; it can accumulate in living organisms and cause serious damage [1]. With the development of industry and modern agriculture, Hg pollution has become a worldwide environmental problem [2], [3]. Numerous cases of mercury pollution in soils have been reported throughout the world [4]. Generally, Hg concentration in unpolluted arable soil is 20–150 µg kg^−1^ [5]. However, the Hg concentration in the soils of paddy fields is much higher, especially in the Philippines and Japan; the average Hg concentration is 24 mg kg^−1^ and 146 mg kg^−1^, respectively, for land irrigated with water contaminated with Hg [6]. The concentration of Hg in paddy soil is as high as 45.9 mg kg^−1^ in some area of China [7].

At high concentrations in soil, Hg can poison plant cells and cause physiological disorders [8], producing detrimental effects on plant growth and metabolism [9], [10]. Hg accumulation in roots prevents the uptake and transport of other mineral nutrients [11], and excess Hg in solution culture can inhibit biomass production [5]. Hg toxicity is not only associated with water uptake and transpiration [12], but also with the decrease in chlorophyll content and photosynthetic efficiency [13]. In addition, Hg stress is believed to trigger the production of reactive oxygen species (ROS), causing oxidative stress and membrane lipid peroxidation in plants [14], [15]. Hg in soil can accumulate in the edible parts of vegetables and crops, and is then transferred to humans via the food chain [16]. Hg is also toxic to humans, causing impaired health in adults. Hg has toxicological effects on the developing central nervous system, on the general physiological systems of children, and adverse effects on the cardiovascular system [17] and fetal brain development [18].

Hg is more easily absorbed and transported in straw and grain than other heavy metals [19]. Previous studies have shown that different genotypes in rice differ widely in their tolerance to Hg toxicity [14]. Wang et al. identified three quantitative trait loci (QTLs) for Hg tolerance in rice seedlings using a recombinant inbred line (RIL) population [20]. Using doubled haploid (DH) lines, three QTLs for Hg accumulation in rice were determined by Yu et al. [14]. Rugh et al. expressed the *merApe9* gene in *Arabidopsis thaliana*, and found that the transgenic seedlings showed low levels of toxicity during the growth and flowering stages because *merApe9* converted toxic Hg^2+^ to the less toxic Hg^0^ [21]. Shen et al. isolated three novel HO genes from rapeseed, and HO-1 has been tested for its ability to regulate plant tolerance to Hg-induced oxidative stress [22]. Wei et al. found that the overexpression of HO-1 confers algal tolerance to excess Hg, whereas silencing of HO-1 had adverse effects on algal growth [23]. Recently, Chen et al. reported that overexpression of the *MTH1745* gene could enhance mercury tolerance in transgenic rice [24]. In maize, there have been some studies on the physiological and biochemical responses to Hg tolerance. The net photosynthesis rate and carboxylation efficiency of maize leaves exposed to 50 ng m^−3^ Hg (in the air) were significantly reduced [25]. *In vivo*, HgCl_2_ inhibited 5-aminolevulinic acid dehydratase activity in excised greening leaf segments of maize, and the inhibition could be alleviated by the addition of KNO_3_ [26]. Additionally, Hg had a strong toxic effect in the roots of maize seedlings, as inferred from the observed higher proportion of oxidized glutathione (GSSG), enhanced carbonyl content and the negative effects on growth [27].

Maize is one of the most important staple crops in the world, and is used as feedstuff and raw-food material in many countries. Maize is used as a staple food for more than 1.2 billion people in sub-Saharan Africa and Latin America [28]. Maize planted in Hg-contaminated paddy soil can accumulate Hg in the grains, which is transferred to consumers by the food chain (soil-plant-human or soil-plant-animal-human), posing a human health risk. However the Hg concentration and distribution pattern in the different tissues of maize and the genetic basis for Hg accumulation remain unclear. Therefore, the objectives of the study were to: (i) examine the accumulation and distribution of Hg in different tissues of maize, (ii) detect QTLs for Hg accumulation in the tissues of maize under Hg-contaminated paddy soil treatment, and to dissect the genetic bases of Hg accumulation in maize.

## Materials and Methods

### The experimental population

A RIL population including 194 inbred lines was used in the study. This population was derived from a cross between two inbred lines, Zong3 and 87-1; the result was an elite hybrid, Yuyu22, which was grown on about 2.7 million hectares per year from 2001 to 2004 in China [29]. The parent Zong3 was selected from a synthetic population with Chinese domestic germplasm; while the inbred line 87-1 was selected from an exotic germplasm. In 2012, the RIL population, both parents and the hybrid were evaluated in Xixian and Changge, Henan Province, China. The experimental materials followed a randomized complete block design with three replications. Each plot included fifteen plants with one 4-m-long and 0.67-m-wide row. The density was 45,000 plants per hectare.

### The mercury content in soil

The experimental materials were evaluated in Xixian country of Xinyang city in Henan province of China (E114°95′–114°72′, N32°35′–32°44′) and Changge country of Xuchang city in Henan province of China (E113°34′–114°08′, N34°09′–34°20′), with an annual average temperature of 15.2°C and 14.3°C; and annual average rainfall of 946 mm and 711.1 mm. The field studies did not involve endangered or protected species, and no specific permissions were required for these locations/activities. The average value of agricultural soil Hg exposure in the Xixian experimental area is 457.57±31.30 µg kg^−1^Hg (pH 6.5), as a result of irrigation with mercury-rich surface water. The average value of agricultural soil Hg exposure in the Changge experimental area is 345.40±22.24 µg kg^−1^Hg (pH 6.5), which was used as a control.

### Quantification of mercury content in the different tissues of maize

Five consecutive plants per row were harvested at physiological maturity stage and at natural withering for mercury content measurements in the different tissues of maize. The whole plant was separated into five tissues: kernels, axis, stems, bracts and leaves, which were ground into powders. A 0.5-g sample of each tissue was digested in 5 ml of HNO_3_/HClO_4_ (80/20 v/v) on a heating block (Digestion Systems of AIM500, A. I. Scientific, Australia). An atomic fluorescence spectrometry (AFS-3000, Beijing Haiguang Analytical Instrument Co, Beijing, China) was used to determine the Hg concentration. Data analyses using the PROC MIXED procedure were performed using SAS 8.0 statistical software.

### Molecular Linkage Construction and QTL Mapping

A total of 263 SSR markers that covered the whole genome of maize were used to construct the genetic linkage map for the RIL population, which spanned 2.361 cM with an average interval of 9 cM between markers [29]. The composite interval mapping method in software QTL Cartographer 2.5 [30] was employed for QTL mapping of measured traits, using the average data of three replications. Model 6 of the Zmapqtl module was used, with scanning intervals of 2 cM between markers and putative QTLs and a 10-cM window. The number of marker cofactors for background control was set by forward–backward stepwise regression with five controlling markers. The logarithm of odds (LOD) threshold was set for each trait by randomly permuting 1,000 times at a significance level of P = 0.05.

## Results

### Hg concentrations in different maize tissues at two locations

**Figure 1:**
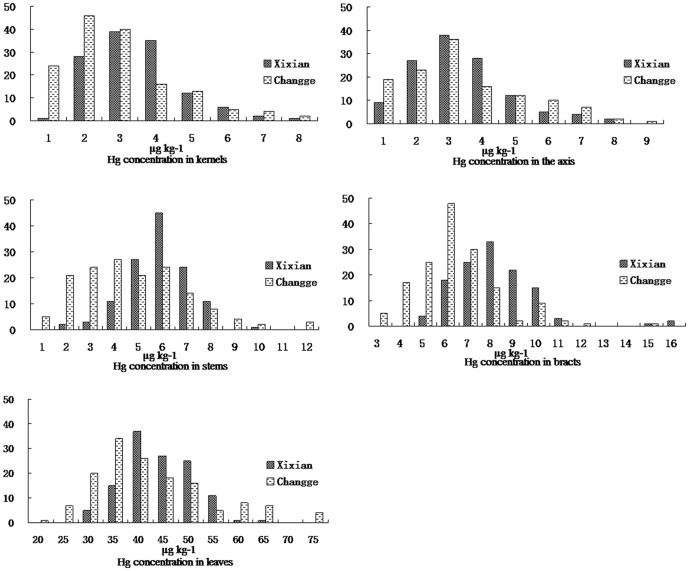
Histogram of Hg concentration in the five tissues of the RIL population.

The Hg concentration in the five tissues varied widely in the RIL population (Table 1, Fig 1). In Xixian, the Hg concentration in the kernels (KHC) for the parent Zong3 was higher (3.53 µg kg^−1^) than that in the parent 87-1 (2.14 µg kg^−1^); the same trend for the two parents was observed in Changge. The KHC was higher in the hybrid Yuyu22 (4.62 µg kg^−1^, 2.74 µg kg^−1^) than in the two parents in both locations. The average KHC of the RIL population was 2.99±1.25 µg kg^−1^ (range 0.73–7.47 µg kg^−1^) and 2.37±1.52 µg kg^−1^ (range 0.26–7.18 µg kg^−1^) in Xixian and Changge, respectively.

**Table 1:** Hg concentration in the five measured tissues in the RIL population.

Location	Population	Trait	KHC (µg kg^−1^)	AHC (µg kg^−1^)	SHC (µg kg^−1^)	BHC (µg kg^−1^)	LHC (µg kg^−1^)
Xixian	P_1_	Mean	3.53	2.89	3.71	8.80	30.20
	P_2_	Mean	2.14	1.51	6.02	6.21	43.20
	F_1_	Mean	4.62	3.40	5.15	5.95	43.83
	RIL	Mean	2.99±1.25	3.84±1.49	5.36±1.30	7.59±1.81	41.06±6.65
		Range	0.73–7.47	1.08–8.37	1.82–9.02	4.07–15.56	27.30–61.45
		Skewness	1.05	0.83	−0.14	1.54	0.28
		Kurtosis	1.51	0.66	0.34	4.99	−0.06
Changge	P_1_	Mean	1.96	1.86	4.70	9.18	43.14
	P_2_	Mean	0.91	3.71	2.83	8.23	37.31
	F_1_	Mean	2.74	3.32	3.67	7.14	45.23
	RIL	Mean	2.37±1.52	2.71±1.76	4.26±2.32	5.83±1.84	39.60±11.79
		Range	0.26–7.18	0.18–8.18	0.47–11.83	2.06–14.03	18.64–74.50
		Skewness	1.11	0.94	0.76	1.11	0.92
		Kurtosis	0.90	0.25	0.56	2.51	0.46

Note: KHC, Hg concentration in kernels; AHC, Hg concentration in the axis; SHC, Hg concentration in stems; BHC, Hg concentration in bracts; LHC, Hg concentration in leaves.

The Hg concentration in the axis (AHC) for the parent Zong3 was higher (2.89 µg kg^−1^) than that of the parent 87-1(1.51 µg kg^−1^) in Xixian. However, in Changge, the value of AHC for the parent Zong3 was lower (1.86 µg kg^−1^) than that of the parent 87-1(3.71 µg kg^−1^). The hybrid had a higher AHC (3.40 µg kg^−1^) in Xixian and a mid-parent value (3.32 µg kg^−1^) in Changge, compared with its parents. The average AHC for the RIL population was 3.84±1.49 µg kg^−1^ (range 1.08–8.37 µg kg^−1^) in Xixian and 2.71±1.76 µg kg^−1^ (range 0.18–8.18 µg kg^−1^) in Changge.

For the Hg concentration in the stem (SHC), the value of the parent Zong3 was lower (3.71 µg kg^−1^) than that of the parent 87-1(6.02 µg kg^−1^) in Xixian, but the opposite trend was found in Changge. The hybrid showed a mid-parent value (5.15 µg kg^−1^, 3.67 µg kg^−1^) at both locations. For the RIL population, the average SHC was 5.36±1.30 µg kg^−1^ (range 1.82–9.02 µg kg^−1^) in Xixian and 4.26±2.32 µg kg^−1^ (range 0.47–11.83 µg kg^−1^) in Changge.

For the Hg concentration in the bract (BHC), the parent Zong3 had higher values (8.80 µg kg^−1^, 9.18 µg kg^−1^) than the parent 87-1 (6.21 µg kg^−1^, 8.23 µg kg^−1^) at both locations. In addition, the BHC (5.95 µg kg^−1^, 7.14 µg kg^−1^) for the hybrid expressed the same low-parent performance at both locations. The average BHC for the RIL population was 7.59±1.81 µg kg^−1^ (range 4.07–15.56 µg kg^−1^) in Xixian and 5.83±1.84 µg kg^−1^ (range 2.06–14.03 µg kg^−1^) in Changge.

The parent Zong3 had a lower Hg concentration (30.20 µg kg^−1^) than the parent 87-1 (43.20 µg kg^−1^) in the leaves in Xixian, but the value of the parent Zong3 (43.14 µg kg^−1^) was higher than the parent 87-1 (37.31 µg kg^−1^) in Changge. The Hg concentration in the leaves (LHC) for the hybrid was 43.83 µg kg^−1^ in Xixian and 45.23 µg kg^−1^ in Changge. For the LHC of the RIL populations, the average value was 41.06±6.65 µg kg^−1^ (range 27.30–61.45 µg kg^−1^) in Xixian and 39.60±11.79 µg kg^−1^ (range 18.64–74.50 µg kg^−1^) in Changge.

Among the different tissues of maize, the kernels had the lowest Hg concentration, followed by the axis, stems, bracts and leaves, and both locations showed the same trend. According to variance analysis, the Hg concentration for the five measured tissues (kernels, axis, stems, bracts, and leaves) in the RIL population exhibited significant variations between genotypes and environments, as well as in the interaction of genotypes and environments (Table 2, P<0.01). For the Hg concentration in five measured tissues in Xixian, the KHC and AHC displayed significant positive relationships, yet the BHC positively correlated with LHC. However, there were no significant relationships among the Hg concentrations in the five measured tissues in the RIL population in Changge by the phenotypic relationship analysis (Table 3, P<0.01).

**Table 2:** Variance analysis of the five measured tissues in the RIL population.

	Kernel	Axis	Stem	Bract	Leave
L	135.49^**^	161.05^**^	59.1^**^	205.2^**^	5.43^*^
G	7.03^**^	2.68^**^	7.17^**^	3.07^**^	6.71^**^
L×G	7.37^**^	2.83^**^	6.46^**^	3.88^**^	8.48^**^

Note: *significant at *P*<0.05, **significant at *P*<0.01.

**Table 3:** Correlation coefficients among five measured tissues in the RIL population.

	Kernel	Axis	Stem	Bract	Leave
Kernel		0.25^**^	0.16	−0.07	−0.03
Axis	0.12		0.04	0.09	0.01
Stem	−0.07	−0.01		0.05	0.05
Bract	−0.05	−0.11	−0.03		0.27^**^
Leave	−0.05	−0.04	0.09	0.15	

Note: **significant at *P*<0.01.

Correlation coefficients in Xixian are above the diagonal, while those in Changge are below the diagonal.

### QTL analysis for Hg concentration in the five tissues of maize

**Figure 2:**
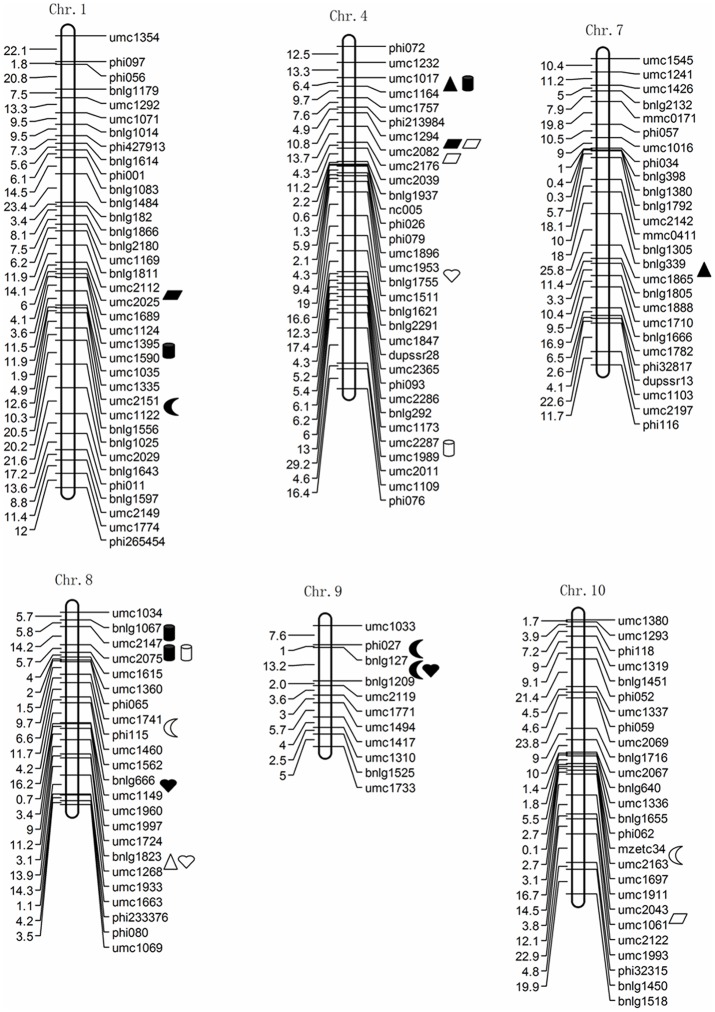
Chromosomal locations of QTLs for Hg concentration for five maize tissues. Note: *Moon* QTL detected for Hg content in kernels, *Triangle* QTL detected for Hg content in the axis, *Cylinder* QTL detected for Hg content in stems, *Heart* QTL detected for Hg content in bracts, *Quadrangle* QTL for Hg content in leaves. Black indicates a QTL detected in Xixian, and lucidity indicates a QTL detected in Changge.

Twenty-three QTLs were detected for Hg concentration in the five tissues of maize in the RIL population at the two locations (Table 4, Fig 2). These QTLs were distributed on chromosomes 1, 4, 7, 8, 9 and 10. Five QTLs for Hg concentration in the kernels were identified in the two environments. In Xixian, there were three QTLs; two of them, *qKHC9a* and *qKHC9b* was adjacent and explained 10.75% and 10.85% of the phenotypic variance, respectively. These two were derived from the parent 87-1. In Changge, two QTL *qKHC8* and *qKHC10* were identified, with 13.00% and 8.42% contribution rate to total phenotypic variance, which came from the parent Zong3 and 87-1, respectively.

**Table 4:** QTLs detected for Hg concentration in five maize tissues.

Location	Tissue	QTL^a^	Location	Flanking-markers	LOD^b^	A^c^	R^2d^
Xixian	kernel	*qKHC1*	238.51	umc2151-umc1122	3.66	0.51	15.11
		*qKHC9a*	9.61	phi027-bnlg127	3.06	−0.42	10.75
		*qKHC9b*	18.91	bnlg127-bnlg1209	2.50	−0.42	10.85
	axis	*qAHC4*	25.81	umc1017-umc1164	2.86	0.44	8.18
		*qAHC7*	145.31	bnlg339-umc1865	3.08	0.70	22.10
	stem	*qSHC1*	211.31	umc1395-umc1590	2.50	0.47	11.76
		*qSHC4a*	30.81	umc1017-umc1164	3.62	0.43	10.94
		*qSHC8a*	6.71	bnlg1067-umc2147	5.88	0.56	18.24
		*qSHC8b*	16.51	umc2147-umc2075	4.20	0.59	20.63
	bract	*qBHC8a*	71.11	bnlg666-umc1149	2.65	0.52	7.80
		*qBHC9*	14.91	bnlg127-bnlg1209	2.85	0.53	8.30
	leave	*qLHC1*	178.51	umc2112-umc2025	2.64	−2.07	9.30
		*qLHC4a*	57.41	umc1294-umc2082	3.19	−3.08	16.46
Changge	kernel	*qKHC8*	46.91	umc1741-phi115	3.18	0.69	13.00
		*qKHC10*	115.61	mzetc34-umc2163	3.16	−0.46	8.42
	axis	*qAHC8*	111.61	bnlg1823-umc1268	2.55	−0.52	6.69
	stem	*qSHC4b*	221.71	umc2287-umc1989	3.76	−0.88	13.94
		*qSHC8b*	25.51	umc2147-umc2075	2.71	0.61	6.70
	bract	*qBHC4*	109.51	umc1953-bnlg1755	2.81	0.53	7.34
		*qBHC8b*	114.61	bnlg1823-umc1268	2.66	0.48	6.44
	leave	*qLHC4a*	59.41	umc1294-umc2082	3.55	4.63	14.38
		*qLHC4b*	69.21	umc2082-umc2176	2.51	3.94	10.39
		*qLHC10*	138.21	umc2043-umc1061	3.38	3.73	9.22

Notes: ^a^QTL detected for Hg concentration in the five maize tissues; ^b^LOD for each QTL; ^c^Additive effect; positive values indicate that Zong3 alleles increase rates; ^d^R^2^, contribution ratio.

For the Hg concentration in the axis, two QTLs, *qAHC4* and *qAHC7* were detected in Xixian and *qAHC8* was detected in Changge, which contributed 8.18%, 22.10% and 6.69% of the phenotypic variance in the Hg concentration in the axis, respectively. The alleles of *qAHC4* and *qAHC7* were derived from the parent Zong3, and the *qAHC8* allele came from the parent 87-1.

Six QTLs associated with SHC were detected at the two locations, which were located on chromosomes 1, 4 and 8. The QTLs contributed 6.70%–20.63% of the total phenotypic variance. Among the QTLs, *qSHC8b* was found at both environments simultaneously, accounting for 20.63% and 6.70% of the total variance, and the effects resulted from the parent Zong3.

Four QTLs were associated with BHC in this study. In Xixian, two QTLs, *qBHC8a* and *qBHC9*, contributed 7.80% and 8.30% of phenotypic variance, respectively. In Changge, the QTLs *qBHC4* and *qBHC8b* explained 7.34% and 6.44% of the phenotypic variance of BHC, respectively. All these QTLs were derived from the high performance parent, Zong3.

For the Hg concentration in the leaves, there were five QTLs at both locations, which explained 9.22%–16.46% of the phenotypic variance. QTL *qLHC4a* was found at both locations simultaneously, and was responsible for 16.46% and 14.38% of the total variance, respectively.

## Discussion

Hg pollution has aroused global concern because of its toxicity to organisms, persistence in the environment and long-range transport [25]. In some contaminated areas, food chain transfer is very common [31]–[34]. In rice, the Hg concentration is much higher in roots than in shoots [14]. Similar results were obtained from other plant species [35], [36]. Meng et al. reported that Hg levels in rice tissues followed the trend: root > stalk> leaf > husk > seed [37], which is consistent with previous studies [38]–[40]. Liu et al. found that the Hg content in the different tissues of maize had features as follows: root > leaf > stalk > grain [41]. In this study, we observed a similar distribution in Hg concentration in different maize tissues (leaves > bracts > stems >axis > kernels). These results demonstrated that the Hg concentration in kernels was lower than that in the other tissues, and the mechanism of Hg accumulation and distribution in different tissues of maize was possibly related to the plant detoxification mechanism. In this study, the Hg concentration in soil in Xixian was higher (457.57±31.30 µg kg^−1^) than that in Changge (345.40±22.24 µg kg^−1^). Compared with the average Hg concentration for the five measured tissues in the two locations, the average value in the RIL population was higher in Xixian than in Changge for all tissues. These phenomena indicated that the Hg concentrations in the different tissues of maize were mainly reflected in the Hg concentration in the soil.

Heavy metals are increasingly becoming environmental concerns because of their release into ecosystems, which pose a threat to human health [23]. Low-cost and ecologically sustainable strategies are needed to restore heavy metal-contaminated soils [28]. However, physical or chemical methods to address heavy metal contamination are environmentally invasive, expensive and inefficient, especially for large scale clean up [42]. Phytoremediation is considered a cost-effective and environmentally friendly approach to remove heavy metals from contaminated soils [43]; however, its application is limited because it is time consuming, there is a lack of suitable plant species and production during remediation is unprofitable [44]. Yu et al. raised the concept of pollution-safe cultivars (PSCs), in which a specific pollutant was accumulated by the edible part at a low level for safe consumption, even when planted in contaminated soil [45]. As an alternative choice, there has been increasing interest in selecting and breeding PSCs [46]. In this study, we found that Hg accumulated in high concentrations in the axis, bracts, stems and leaves, which represent the main biomass products in maize, while the kernels contained the lowest concentration among the five tested tissues. For KHC in the RIL population, two parents and the hybrid, the highest value was 4.62 µg kg^−1^ in Xixian and 2.74 µg kg^−1^ in Changge; however, the values were much lower than 20 µg kg^−1^, which is the maximum recommended by the Chinese National Standard Agency [47]. Maize is the most commonly planted crop worldwide, with broad adaptability. Thus, there is the potential to decrease the concentration of Hg in Hg-contaminated paddy soil, and maintain maize production simultaneously by selecting and breeding maize Hg PSCs.

There are many forms of Hg in soil, including the elemental (Hg^0^), ionic (Hg^2+^), hydroxide (Hg(OH)_2_), methyl (MeHg), and sulfide (HgS) [48], [49]. The environment and the genotype control the uptake of Hg species in rice [50], and rice varieties differ widely in their absorption and tolerance of Hg toxicity [14]. In peas, six Hg^2+^ responsive genes were identified using suppression subtractive hybridization [51]. Their gene products were involved in the salicylic acid (SA) biological defense system, the biosynthetic pathway of isoflavonoids, antioxidant reactions, sulfur metabolism, and cell wall rigidity [51]. Heidenreich et al. profiled the transcriptome of *A. thaliana* exposed to Hg^2+^, and identified Hg-induced genes that encoded proteins involved in chlorophyll synthesis, cell wall metabolism, and P450-mediated biosynthesis of secondary metabolites [52]. In addition, Yamaguchi et al. found that Hg and other heavy metals could induce certain genes at the same time [53]. Recently, several other genes related to Hg accumulation have been identified [54], [55]. In rice, 20 proteins that were differentially expressed in roots under Hg treatment were identified using 2-D electrophoresis. They were involved in cellular functions, including redox and hormone homeostasis, chaperone activity, metabolism, and transcription regulation [56]. Wang et al. identified three QTLs for relative root length in rice under Hg stress, which coincided with QTLs for drought, Zn^2+^, Fe^2+^, Cd^2+^, Cu^2+^ and Al^3+^ toxicity tolerance in rice [19]. In this study, out of the 23 QTLs for Hg concentration in different maize tissues, three (*qKHC9a*, *qKHC9b*, *qBHC9*) were adjacent to two QTLs for drought tolerance using the F_2:3_ populations derived from the same parents Zong3 and 87-1 [57]; one QTL, *qLHC1*, was in linkage with two QTLs for arsenic accumulation using RIL populations derived from a cross between two maize inbred lines, Huang-C and Xu178 [58]. These results suggested that the QTLs related to Hg accumulation had pleiotropic effects.
